# High Mortality in the Male Breast Cancer Community: A Report on Its Drivers and a Demand for Change in Healthcare Policy

**DOI:** 10.7759/cureus.100141

**Published:** 2025-12-26

**Authors:** Srikavya Pasumarthy, Peggy Miller, Cheri Ambrose, Patricia Washburn, Lopamudra Das Roy

**Affiliations:** 1 Medicine, Western Michigan University Homer Stryker M.D. School of Medicine, Kalamazoo, USA; 2 Oncology, Breast Cancer Hub, Concord, USA; 3 Oncology, Male Breast Cancer Happens, Prairie Village, USA; 4 Oncology, Male Breast Cancer Global Alliance, East Hanover, USA

**Keywords:** early detection, healthcare policy and management, male breast cancer, mortality, screening

## Abstract

Introduction: Male breast cancer (MBC) is a rare cancer with a high mortality rate. MBC remains a rare but significant health concern in the United States, with ongoing cases and associated mortality. This mortality rate could be attributed to late detection in men as a result of a lack of awareness, gender bias, and stigma around sexual health.

Aims: In relation, the goals of this study are threefold: (i) to shed light on the role late detection may play in mortality in MBC patients; (ii) to highlight the epidemiological factors that contribute to MBC; and (iii) to promote early detection.

Methods: Questionnaires were created and circulated to MBC patients in the United States and their families to garner information on their disease progression. This study was conducted remotely, and data were received from 122 patients who were part of the MBC coalition (now two separate organizations - Male Breast Cancer Happens and Male Breast Cancer Global Alliance).

Results: A total of 41 (85.30%) survey takers had a grade 1 or 2 cancer, indicating that most cancers were slow-growing. However, it was also found that 52 (68%) men were diagnosed at stage 4, indicating delayed detection.

Conclusion: For a cancer so rare, it is easy for the poor outcomes that plague those impacted by MBC to go unnoticed. The lack of awareness and screening for MBC leads to late detection and death. Therefore, it is imperative that the system practices screening protocols that promote early detection, thereby changing the current healthcare policy.

## Introduction

Male breast cancer (MBC), unlike female breast cancer (FBC), is an extremely rare form of cancer. While it is projected that 316,950 new cases of FBC will be diagnosed in the United States (US) in 2024 [1], only 2,800 MBC cases are predicted to be diagnosed [2]. Thus, MBC makes up less than 1% of the breast cancer cases in the US.

While FBC cases largely outnumber those of MBC, it is unclear whether the diseases are really all that similar. Currently, research suggests that both similarities and dissimilarities exist between MBC and FBC. For example, BRCA1 and BRCA2 mutations predispose both men and women to breast cancer. In men, BRCA2 mutations are more likely to predispose men to breast cancer [3], whereas in women, BRCA1 mutations may be comparable to BRCA2 mutations in predisposing women to breast cancer [4].

Another similarity that can be drawn is the cancer markers used to classify the two cancers. MBC, like FBC, can be classified by hormone-positive markers. Specifically, MBC can be estrogen receptor positive (ER+), progesterone receptor positive (PR+), and HER2-positive. For a cancer to be ER+ or PR+, the cancer must respond to estrogen or progesterone, respectively, to grow [5]. ER+ or PR+ cancers are also commonly referred to as hormone-positive cancers. HER2-positive cancers, on the other hand, are so named because these cancers are faster growing in the presence of the HER2 protein. These cancers are also far more likely to respond well to therapies that target HER2 proteins, such as trastuzumab [6]. These are targeted markers in FBC for treatment, which have been translated into their use for MBC treatment.

Many dissimilarities exist as well. For instance, men have been found to have a higher median age at diagnosis compared to women [7]. Another significant difference between MBC and FBC is that the prevalence of the BRCA1 and BRCA2 mutations varies by sex - there is an increased prevalence of the BRCA2 mutation in MBC when compared to FBC [8].

These are a few of the many differences that exist between MBC and FBC. However, despite these glaring differences between the two cancer types, MBC and FBC are treated quite similarly in the clinic. In fact, treating MBC like it is FBC can prove to be less effective for patients with MBC.

For example, consider aromatase inhibitors and tamoxifen. These two treatments are standard for FBC treatment. When these treatments are administered to men, they show varying outcomes. According to one study, tamoxifen, when administered for MBC, showed similar survival rates (89.2%) to FBC (85.1%) [9]. Although the survival rate between men and women on tamoxifen may be similar, tamoxifen overall decreases the chance of recurrence in men. One study that evaluated MBC relapse rates in relation to tamoxifen found that men who received tamoxifen had a recurrence rate of 11.2% with tamoxifen treatment and 18.2% without treatment. Aromatase inhibitors, however, showed different survival rates and poorer prognosis in men. While FBC patients who received aromatase inhibitors had a better survival rate (85.0%), patients with MBC who received aromatase inhibitors had a lower survival rate (73.3%). This difference was noted to be statistically significant (p=0.028) [10].

It is important to note that the rarity of MBC precludes the generation of sufficient research on the topic. As MBC remains largely underexplored in research, this work seeks to contribute to the expanding body of literature evaluating the disease while underscoring the importance of earlier detection and screening. Moreover, it aims to raise awareness within both the research community and the public regarding the need for improved and timely interventions for MBC.

This paper has three primary objectives: (i) to evaluate the role of late detection in MBC-associated mortality; (ii) to identify epidemiological and demographic factors contributing to MBC; and (iii) to assess the impact of awareness and early detection programs on patient outcomes.

## Materials and methods

This study was completed remotely by the research team at Breast Cancer Hub, Concord, CA, a nonprofit organization. The data collection for the study ran from April 2021 to September 2021. The analysis of these data was completed. The WCG Institutional Review Board (IRB) protocol number is 20204167, and the IRB study number is 1322112.

Two main surveys were created in this study [11]. The first survey was a comprehensive questionnaire that was directed towards patients who experienced MBC in any capacity. Through this survey, 48 patients completed the questionnaire. Questions on the survey were targeted to obtain information on the lifestyle factors, family history, past medical history, and demographic information on patients. Questions were structured in multiple-choice or short-answer format. Additionally, questions that addressed the clinical presentation and characteristics of the cancer were asked as well, including the grade, stage, and age at diagnosis. The purpose of these questions was to identify what factors increased the risk of developing MBC. These questions also helped identify if late detection was a trend in the MBC population. Questions that addressed certain well-known factors that often increased the risk of MBC were asked; topics such as hormone status, genetics, tobacco consumption, and others were included. Questions consisted of those that sought out open-ended descriptive answers to limit bias.

Additionally, a second separate survey was created to address patients whose cancer had relapsed. The parameters were essentially the same for both surveys; however, while the first survey addressed the initial onset of cancer, the relapse survey’s questions were targeted to inquire into the characteristics of the relapsed cancer and what factors might have contributed to the relapse.

The questionnaire was developed on Squarespace and was ultimately available for patient use. This questionnaire was broken up into multiple sections, including questions that inquired about lifestyle, hormone, receptor status, experience with screening, stage of cancer, grade of cancer, etc. The formatting for answer choices varied between multiple-choice and short answer, depending on the level of detail required for the question. Please see the references section for the fourth questionnaire. Descriptive statistics were used in the analysis of the data. The mean, median, mode, and standard deviation were used as measures to determine the distribution of the data. The survey was then circulated to MBC patients who were a part of two other organizations based in the US. These organizations also provided access to the MBC population data from 77 patients whose lives were lost to MBC. Some of the data that were provided included the age at diagnosis, stage at diagnosis, grade of cancer, and age at death of various patients.

Answers from all the datasets and surveys were then promptly filtered through for relevant, quantitative information (e.g., age, grade, stage) and then charted on Google Sheets. Each of these datasets' datapoints was classified based on what kind of stage the cancer was, or what kind of grade the cancer was, and these numbers were then converted to percentages of the total set and further converted into graphs using the inherent statistical features of the software. If the data were largely numerical, then the data were analyzed in the form of calculated means, medians, modes, and standard deviations.

For the purposes of publication, the data were completely de-identified, and the names of survey takers were kept anonymous. The research scholar analyzed the dataset, and the data and analysis were validated by the principal investigator and co-authors of the project.

Incomplete responses were excluded from the dataset. Inclusion criteria included data from individuals who were determined as male at birth who are or were fighting breast cancer. Data were either obtained directly from patients or their family members if patients were unable to complete it themselves or had deceased.

## Results

This study accrued data on a total of 122 MBC patients significantly sized dataset considering the rarity of the cancer. The sources of this data are broken down as follows: 77 data points were obtained from the dataset of the deceased patients provided by two collaborating organizations; 45 of the data points were obtained from live MBC patients who took the survey; and three data points were obtained from patients whose family members filled out the same survey.

**Figure 1:**
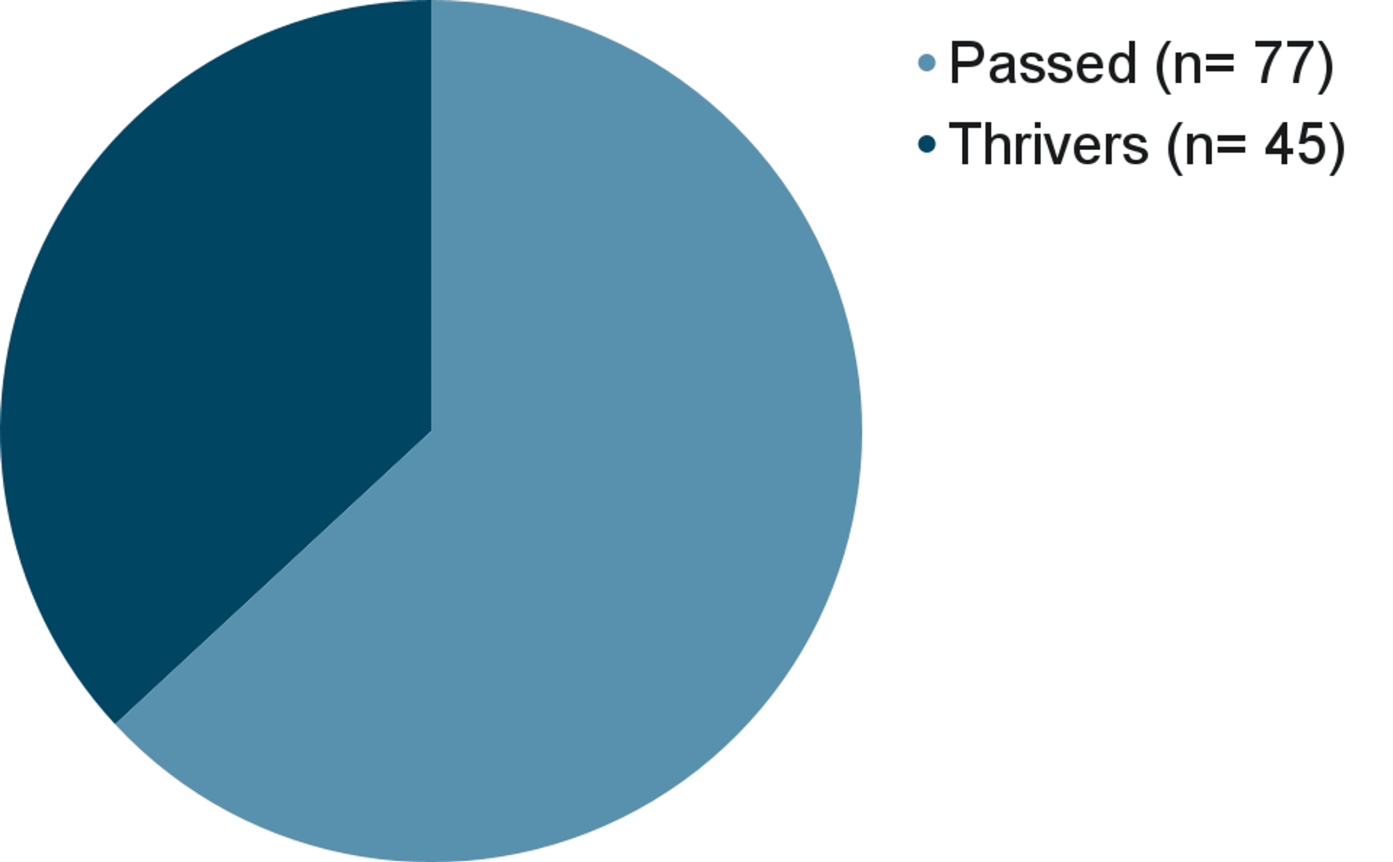
Percent of lives lost to male breast cancer.

The most incidental finding that can be reported here is the high mortality in MBC patients. Among both our survey takers and the dataset of deceased patients, a mortality rate of 77 (63.1%) is due to MBC. See Figure 1.

**Figure 2:**
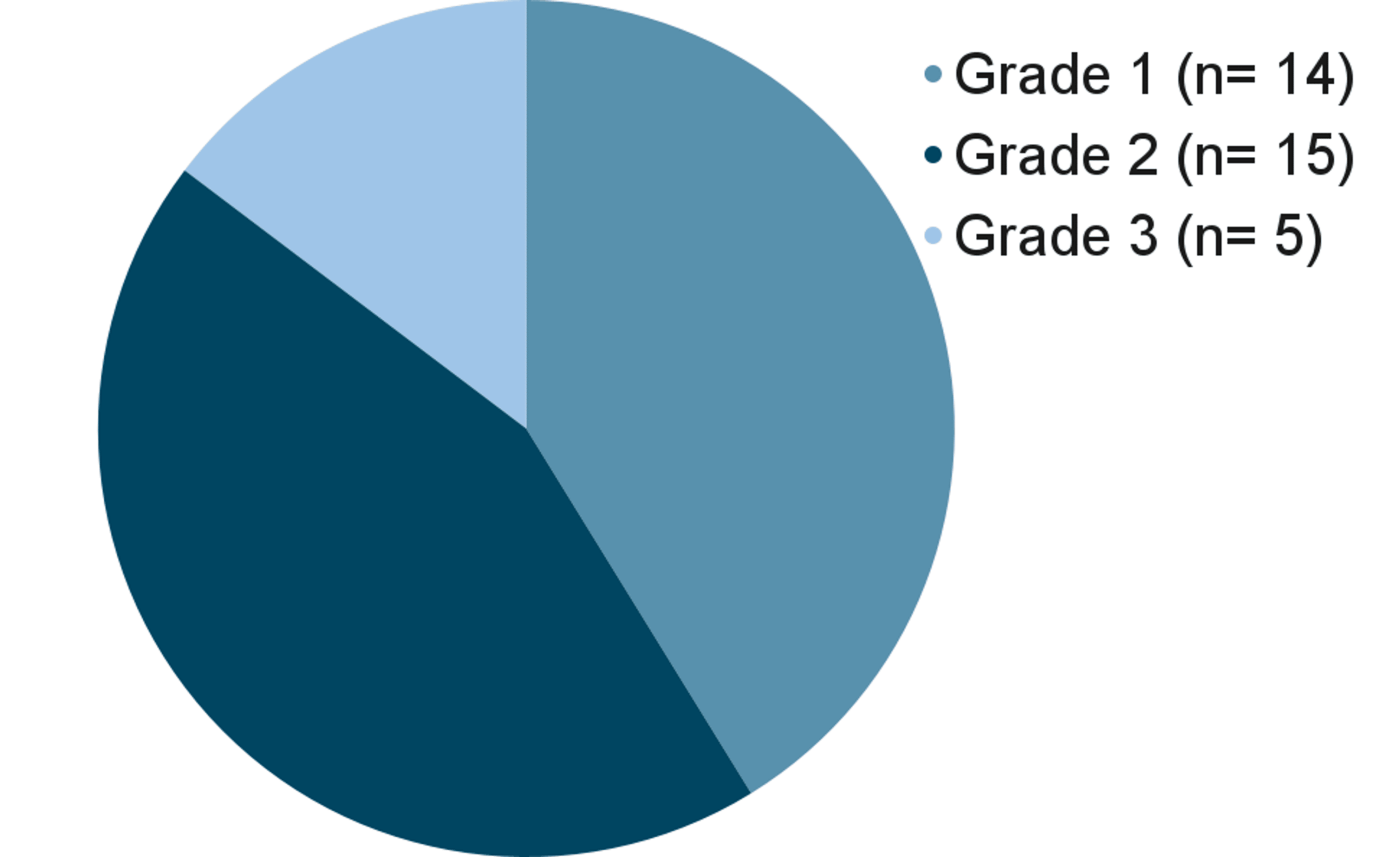
Distribution of cancer grades in patients with male breast cancer.

Furthermore, analysis of the survey exhibited the following statistics: men are diagnosed with MBC at the age of 54 years on average (N=48, std dev.=10.71). Regarding the grade of cancer among survey takers, 14 (41.18%) had grade one, 15 (44.12%) had grade two, and five (14.71%) had grade three MBC (n=34). See Figure 2 for a visual representation. Additionally, 42 (93.33%) of patients had hormone-positive cancers (n=45).

The findings above highlight something very important: the high mortality of MBC and the low grade of such cancers imply that MBC-related mortality can be reduced. Low-grade cancers are often slower growing and less pleomorphic, indicating better prognosis and a larger window for early detection. However, the mortality rate for MBC is still very high. This is because MBC is not screened early enough, if at all. In fact, this survey showed us that only 40 (8.33%) patients reported detection through clinical breast exams. Clinical breast exams are not a part of the protocol for men’s physicals, indicating that these cancers were perhaps found accidentally or due to suspicious findings.

Detection of MBC often occurred due to breast self-exam instead. In fact, 30 (62.50%) of our survey takers reported that their cancer was identified through breast self-exam. However, a large portion of the 30 (62.50%) indicated that their detection of the lump was accidental, making this finding misleading. These statistics tell us one key thing: the lack of clinical MBC screening predisposes men to be far more susceptible to late detection. Therefore, breast exams need to be incorporated into physicals for male patients.

Furthermore, many contributing lifestyle and genetic factors to MBC were found in this study. Out of 48 survey takers, 28 (58.33%) consumed alcohol, 16 (33.33%) consumed tobacco, and nine (18.75%) consumed both tobacco and alcohol. Furthermore, 23 (48.89%) of patients were obese (BMI >30), 19 (40.00%) were overweight (BMI 25.0-29.9), and five (11.11%) were of normal weight (BMI 18.5-24.9). Additionally, 29 (60.42%) patients report a family history of cancer, with 22 (45.83%) reporting ovarian, breast, or prostate cancer in their lineage. It was also found that 31 (64.58%) suffered from a form of chronic illness connected to inflammation. Additionally, nine (18.75%) survey takers tested positive for either BRCA1 or BRCA2 mutations, and 42 (87.5%) of patients’ cancers were hormone-positive.

**Figure 3:**
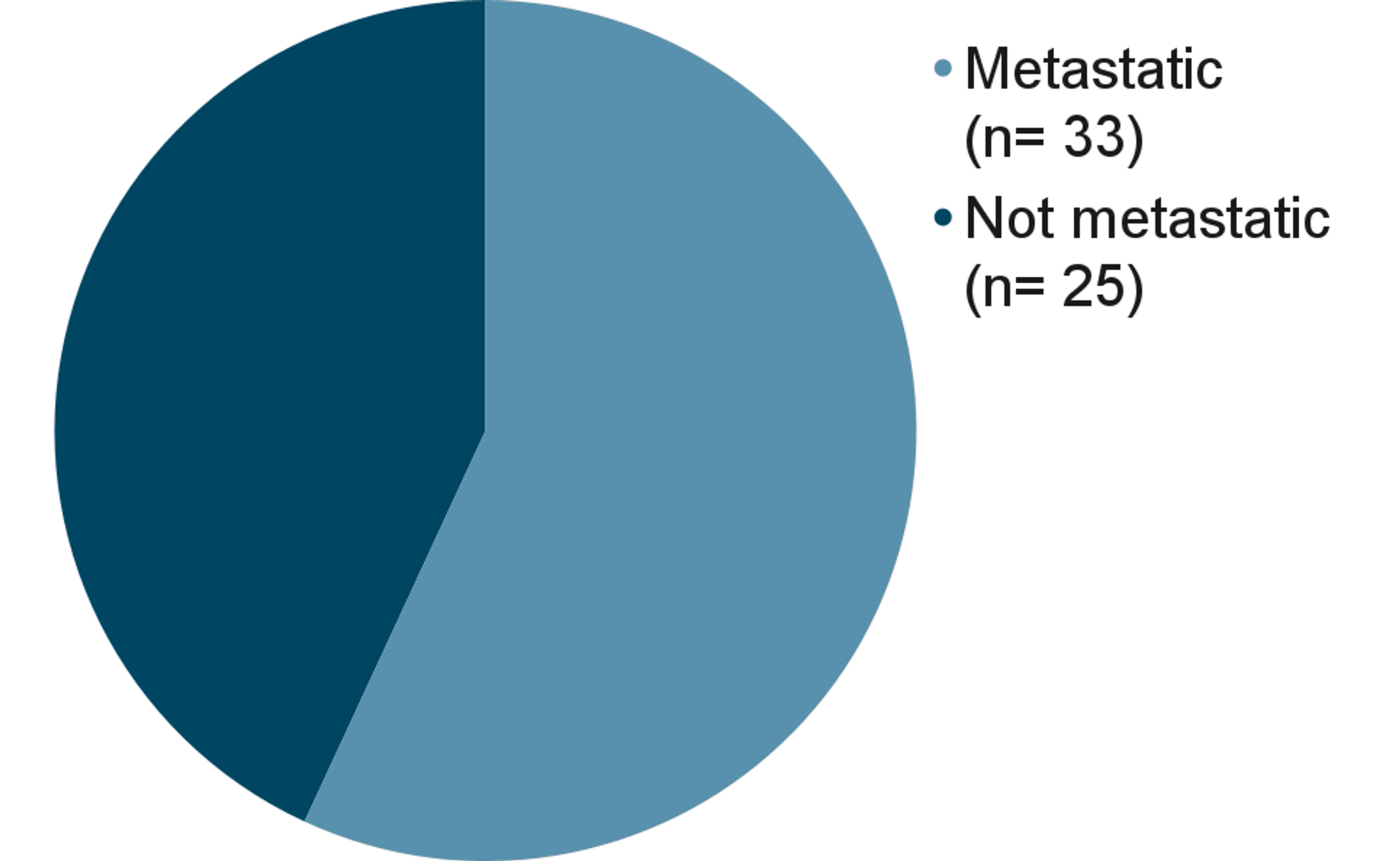
Percent of patients in our dataset that had metastatic breast cancer.

In addition, analysis of the survey and the dataset of deceased patients yielded the following results: 58 out of 122 patients in the dataset declared their stage at diagnosis. Out of these 58 patients (33 alive and 25 deceased), 33 (56.00%) of patients were stage four (see Figure 3). Furthermore, out of the 25 deceased patients whose loved ones provided their stage at diagnosis, 17 (68.00%) were stage 4 at diagnosis.

Finally, out of the 48 survey takers, five (10.42%) patients’ cancer relapsed. All five of these patients had hormone-positive cancers, and only one of these patients took tamoxifen as a treatment.

## Discussion

There are a few alarming findings that this study highlights, but there are also potential solutions. Notably, the median age at diagnosis for men with breast cancer is 54 years, which is younger than the existing literature, which states that the median age of MBC at diagnosis is 68 years [12] and 62 years for FBC in the US [1]. Therefore, this study suggests that men present with breast cancer much earlier than previously reported in existing literature [7].

A lower median age at diagnosis calls for the need for earlier detection, but it is not the only reason why earlier detection is so important in men. Our results have shown that 52 (68%) patients from the deceased cohort had stage four cancer. Studies have supported that MBC is often caught after metastasizing more frequently than FBC [13,14]. These statistics highlight that MBC, when caught, is often diagnosed at an advanced metastatic stage. It is also important to note that MBC is often lower in grade, with 14 patients (41.18%) presenting with grade one cancer and 15 patients (44.12%) presenting with grade two cancer in our study. This finding has been supported by previous literature, which also states that MBC is typically lower in grade but caught at a higher stage [14]. These findings, in conjunction, declare that, while MBC is slow-growing, it is also caught at stage four quite often, showing that these cancers have been neglected and unscreened, leading to poor outcomes.

While current guidelines recommend initiating FBC screening between the ages of 40 and 45, screening for MBC should begin earlier, given the data of this study. Additionally, physicians should conduct routine clinical breast exams on male patients starting at age 18. Similar guidelines have been proposed by the National Comprehensive Cancer Network (NCCN), which suggests that men with BRCA1 or BRCA2 mutations should begin clinical breast exams at age 35 or be taught how to perform breast self-exams [15]. In relation, this study aims to take the NCCN's recommendations a step further and advocate for screening earlier and not restrict the protocol to just BRCA mutation carriers. In this dataset, only nine patients (18.75%) were BRCA1- or BRCA2-positive, while the remaining 39 (71.25%) patients did not have these mutations. Though BRCA mutations overall increase the risk of breast cancer in men, supporting prior research that found men with BRCA mutations are more likely to develop MBC [3]. As is well-established, BRCA1 mutations increase breast cancer risk in women [16]. This additionally underscores the shared risk factors between MBC and FBC.

Furthermore, these suggested guidelines do not have to and should not be restricted to the US. Many regions globally, including parts of the US, lack access to adequate healthcare. According to the WHO, 4.5 billion people do not have access to essential health services [17]. This is why public awareness around monthly breast self-exams is important.

While early detection and increased screening are central to this paper, this study also sought to explore factors that may be associated with MBC. This research indicates that obesity and chronic inflammatory conditions, such as arthritis and gout, may correlate with MBC. Thirty-one (64.58%) survey takers suffered from a form of chronic illness connected to inflammation. Several papers have already established that arthritis and inflammatory conditions are associated with breast cancer [18-22]. These findings highlight that inflammation could be associated with the development of MBC.

Some limitations of this study should be noted. The sample size (n) is relatively small, though it is important to note that the rarity of the disease made increasing the sample size challenging. A smaller sample may impact the robustness of these findings, though every effort was made to collect as large a dataset as possible.

## Conclusions

MBC is a disease with a multifactorial etiology with strong associations with both lifestyle and genetic factors. Therefore, maintaining a healthy lifestyle is prudent for prevention. Additionally, this disease can occur at any age and is often underdiagnosed or caught too late, leading to high mortality rates. It is important to recognize, however, that the high mortality of MBC is avoidable. Most of the cancers that have been diagnosed according to this dataset were slow-growing and of an advanced stage, indicating that these cancers had been developing for a while, and simply screening earlier could lead to more lives saved and a better prognosis overall. Currently, there is no universalized effort to screen for MBC via clinical breast exam, mammograms, etc. However, it is important to note that this screening is not just an individual effort. This is a true public health need, and as such, systemic and community-level interventions are necessary: healthcare providers should incorporate MBC awareness and clinical breast exams into routine physicals. Only then can earlier detection be promoted at the level of the population. It is important to note that the slow-growing rate of MBC, as revealed by this paper, makes early detection and screening momentous prospects in preventing mortality (of note, this study is limited by a smaller sample size, self-reported data, and limited geographic diversity), highlighting the need for it. While screening for MBC is one component, public health officials should also raise awareness regarding the presence of MBC and the similarity in mortality rates between male and FBC to lessen stigma, thus expectedly improving the desire to obtain screening and hopefully producing a domino effect to decrease mortality. To summarize, this system must be revolutionized in a multifactorial way through regular in-office clinical breast exams for men, increased education in the community through education programs or efforts, and encouragement to improve lifestyle and diet in men. This approach may decrease mortality in the MBC community and promote early detection.
